# Time-synchronized immune-guided SBRT partial bulky tumor irradiation targeting hypoxic segment while sparing the peritumoral immune microenvironment

**DOI:** 10.1186/s13014-019-1423-9

**Published:** 2019-12-04

**Authors:** Slavisa Tubin, Martin Ashdown, Branislav Jeremic

**Affiliations:** 1 KABEG Klinikum Klagenfurt, Institute of Radiation Oncology, Feschnigstraße 11, 9020 Klagenfurt am Wörthersee, Austria; 2 Melbourne, Australia; 3 BioIRC, R&D Center for Biomedical Research, Kragujevac, SERBIA, Research Institute of Clinical Medicine, 13 Tevdore Mgvdeli St., 0112 Tbilisi, Georgia

**Keywords:** Immune-guided timing, Partial irradiation, Bystander effect, Abscopal effect, Tumor hypoxia, Immune microenvironment

## Abstract

**Background:**

A novel unconventional SBRT-based PArtial Tumor irradiation targeting HYpoxic clonogenic cells (SBRT-PATHY) for induction of the tumoricidal bystander (BE) and abscopal effects (AE) was developed by translating our preclinical findings to a clinic in 2016. In order to further improve BE/AE response rate, SBRT-PATHY was upgraded in 2018 by the sparing of peritumoral immune microenvironment as a new OAR, defined by its own dose-constraints. Considering the anti-tumor immune response homeostatic fluctuation, which is cyclically suppressed and incited (“switched off and on”), we synchronized SBRT-PATHY with its most excitable phase, in order to overcome tumor tolerance locally and systemically. The aim of this study, therefore, was to report on the initial results of our latest innovation aimed to further improve BE/AE response rate by testing the effectiveness of the time-synchronized immune-guided SBRT-PATHY.

**Materials and methods:**

In order to serially map the homeostatic anti-tumor immune response-fluctuations, High Sensitive C-Reactive Protein (HS-CRP), Lactate Dehydrogenase (LDH) and Lymphocyte/Monocyte Ratio (LMR) were analyzed using high-order polynomial trend analysis as surrogate of immune system response. After the biomarker data analysis detected the immune fluctuations and related idiosyncratic immune cycle periodicity, we determined the “most favourable” and “least favourable” treatment time-positions in the immune cycle. In order to evaluate the impact of an idiosyncratic immune cycle on treatment outcomes, our first consecutive four patients were treated on the “most favourable” while the remaining four on the “least favourable” day.

**Results:**

The median follow-up was 11.8 months. The biomarker data analysis showed periodic immune response fluctuations of regular frequency. The “right” synchronization of SBRT-PATHY with the “most favorable day” of anti-tumor immune response was accompanied with improved clinical outcomes in terms of BE/AE-response rate.

**Conclusion:**

We believe the right synchronization of radiotherapy with the homeostatically oscillating immune response may improve the probability of inducing BE/AE.

Present study has been retrospectively registered on 18th of October 2019 by the ethic committee for Austrian region „Kärnten “in Klagenfurt (AUT), under study number A 37/19.

## Introduction

Clinical exploitation of the bystander (BE) and abscopal effects (AE) was an objective of our long-standing translational oncology research aimed to overcome the outcome-limiting factors related to the unresectable bulky tumors. Despite the developments in oncological therapy BE/AE remain still rare phenomena [1].

In order to improve the therapeutic-ratio by exploiting BE/AE an unconventional partial tumor irradiation targeting the hypoxic segment was developed in 2016 in our institute [2]. Our preclinical findings indicated that the hypoxic in respect to normoxic tumor cells, if selectively irradiated as inductor of BE/AE, show higher potential for the generation of BE/AE [3]. The subsequent translation of these findings to a clinic led to the introduction of a novel **SBRT**-based **PA**rtial **T**umor irradiation targeting **HY**poxic clonogenic cells (**SBRT-PATHY**) showing promising BE/AE-response rates [2, 4]. Recently, the Italian group confirmed efficacy of SBRT-PATHY in their initial experience [5]. Considering the immune-mediated nature of BE/AE and in order to further improve BE/AE response rate, SBRT-PATHY was upgraded in 2018 by the sparing of peritumoral immune microenvironment as a new OAR, defined by its own dose-constraints [4, 6]. Our concept implied that for successful therapeutic immune modulation, the entire tumor volume may not need to be irradiated but only a part of the tumor. This should initiate antigen shedding, increase effector T cell activation and lead to favorable alterations in radiation-spared peritumoral immune environment [7].

Currently, some studies have described an association between the radiation-induced lymphopenia with poor oncologic outcome, indicating that radiotherapy using large volumes and multiple daily fractions can lead to immunosuppression [8, 9]. On the other side, some studies have shown potential therapeutic benefits by eventual ablation of regulatory (“suppressor”) T cells with limited (single-dose) systemic therapies [10–12] given “at the right time” in order to selectively ablate those suppressor T cells while sparing the effector T cells. Thus, suggesting that the accurate timing of limited therapy may play a major role in treatment efficacy. Following the recent reports of the anti-tumor immune response oscillating over several days [13–16], we hypothesized the following: by monitoring before the treatment immune-specific biomarkers as the surrogates of homeostatically fluctuating immune response, which is cyclically suppressed and incited (“switched off and on”), it would be possible to determine a periodicity of immune response and, based on that, to synchronize SBRT-PATHY with its most excitable phase, in order to overcome tumor tolerance locally and systemically.

The objective of this study, therefore, was to report on the initial results of our latest innovation aimed to further improve BE/AE response rate by testing the effectiveness of the time-synchronized immune-guided SBRT-PATHY.

## Materials and methods

### Timing of SBRT-PATHY with respect to an underlying fluctuating anti-tumor immune response

**Fig. 1:**
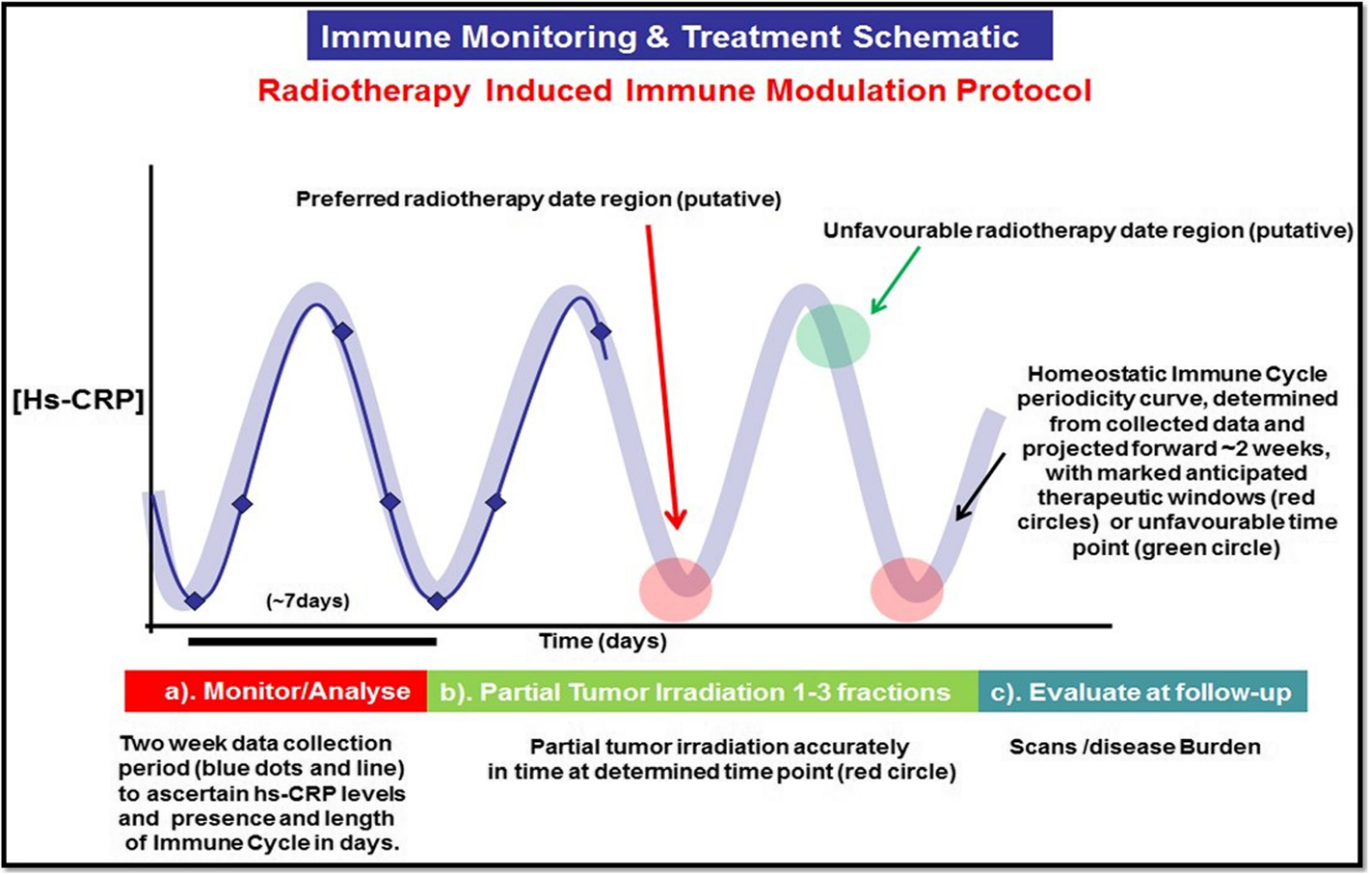
Radiotherapy induced immune modulation protocol schematic: this figure describes the three phases of the timing of SBRT-PATHY protocol as: (**a**) the monitoring, collection and analysis of serial data to establish the presence of immune cycle and its periodicity (blue dots and line), (**b**) projection forward to the putative “most favorable time-position in a cycle” (red circles) or “less favorable time-position“(green circle) to initiate radiotherapy, (**c**) evaluate at follow-up via scans to provide the evidence for patient responses stratification determined by timing of the treatment

**Fig. 2:**
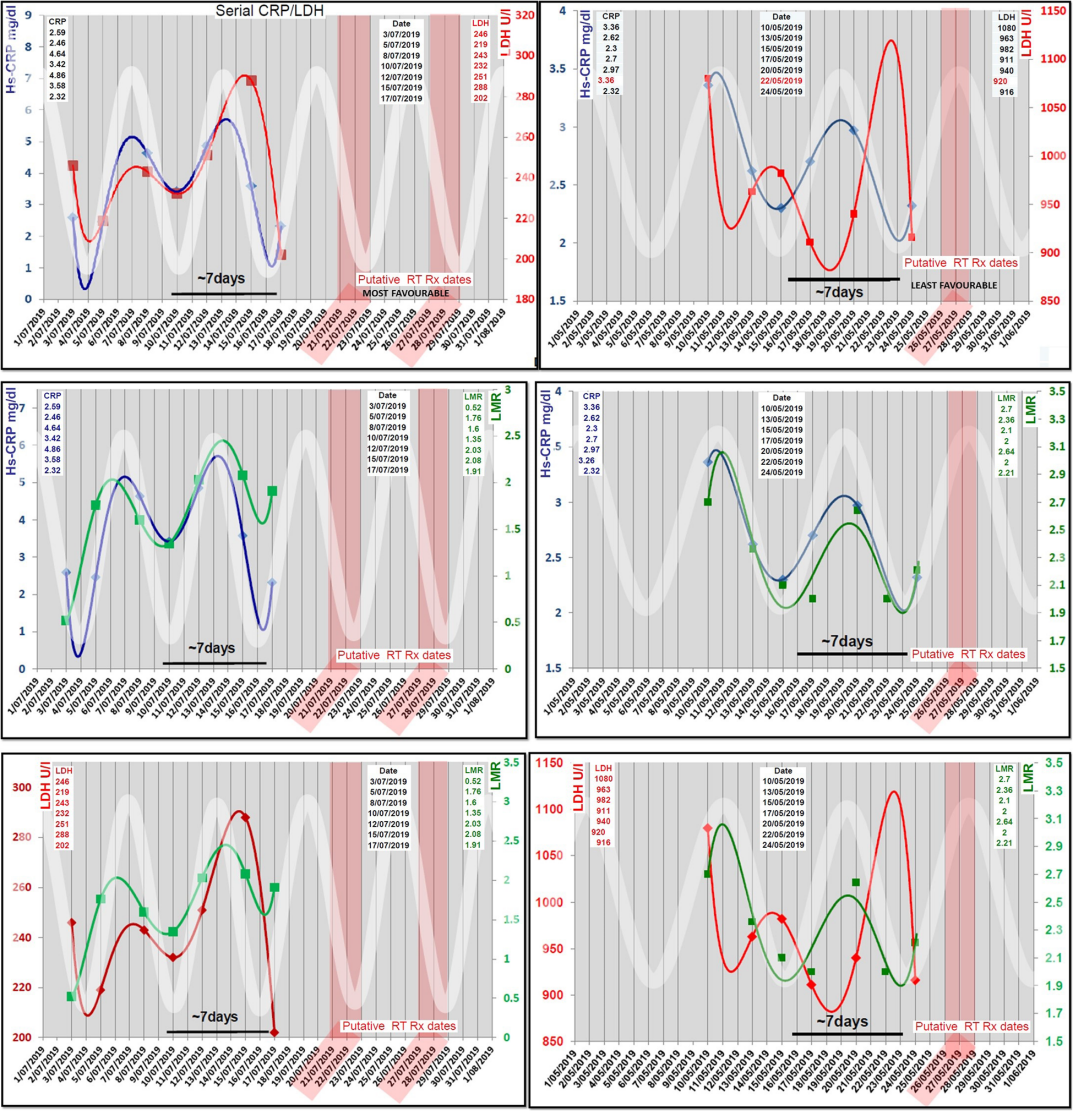
Immune oscillation monitoring and treatment profile: This figure describes the graphical representation of serial monitoring data over 2 weeks of two example patients in left and right columns. Each graph panel compares the polynomial cyclical relationship and periodicity of biomarkers Hs-CRP/ LDH, Hs-CRP/LMR and LMR/LDH against a standard sine-wave (thick white line). This white sinusoidal line projects forward in time in attempt to identify the putative “most favourable” or “least favourable” dates to treat (marked as red boxes)

Two weeks prior to initiation of SBRT-PATHY, serial (7x) bloods were taken from each patient every second day and assayed for serum biomarkers such as *High Sensitive C-Reactive Protein* (HS-CRP), *Lactate Dehydrogenase* (LDH) and also *Lymphocyte/Monocyte Ratio* (LMR). Data from the assays were analysed to define cyclical fluctuations using high-order polynomial trend analysis. In order to determine each patient’s idiosyncratic immune cycle periodicity, the trend/periodicity analysis was performed to generate a standard sine wave of similar periodicity (over several cycles) and visually overlayed and aligned (peak & trough) on the generated polynomial trend graph. This alignment was done in order to project forward in time to the designated/putative treatment date(s) (Figs. 1 and 2). After the biomarker data analysis detected the immune fluctuations and related idiosyncratic immune cycle periodicity, we determined the “most favourable” and “least favourable” treatment time-positions in the immune cycle. As the biomarkers we used are known to rise and fall periodically over several days with the initiation and then homeostatic termination of the immune response [13, 17], we defined a pre-trough time-position as the “most favourable” treatment phase in the immune cycle, while a pre-peak region as the “least favourable” day for SBRT-PATHY. In order to evaluate the impact of an idiosyncratic immune cycle on treatment outcomes, our first consecutive four patients were treated on the “most favourable” while the remaining four on the “least favourable” day.

**Fig. 3:**
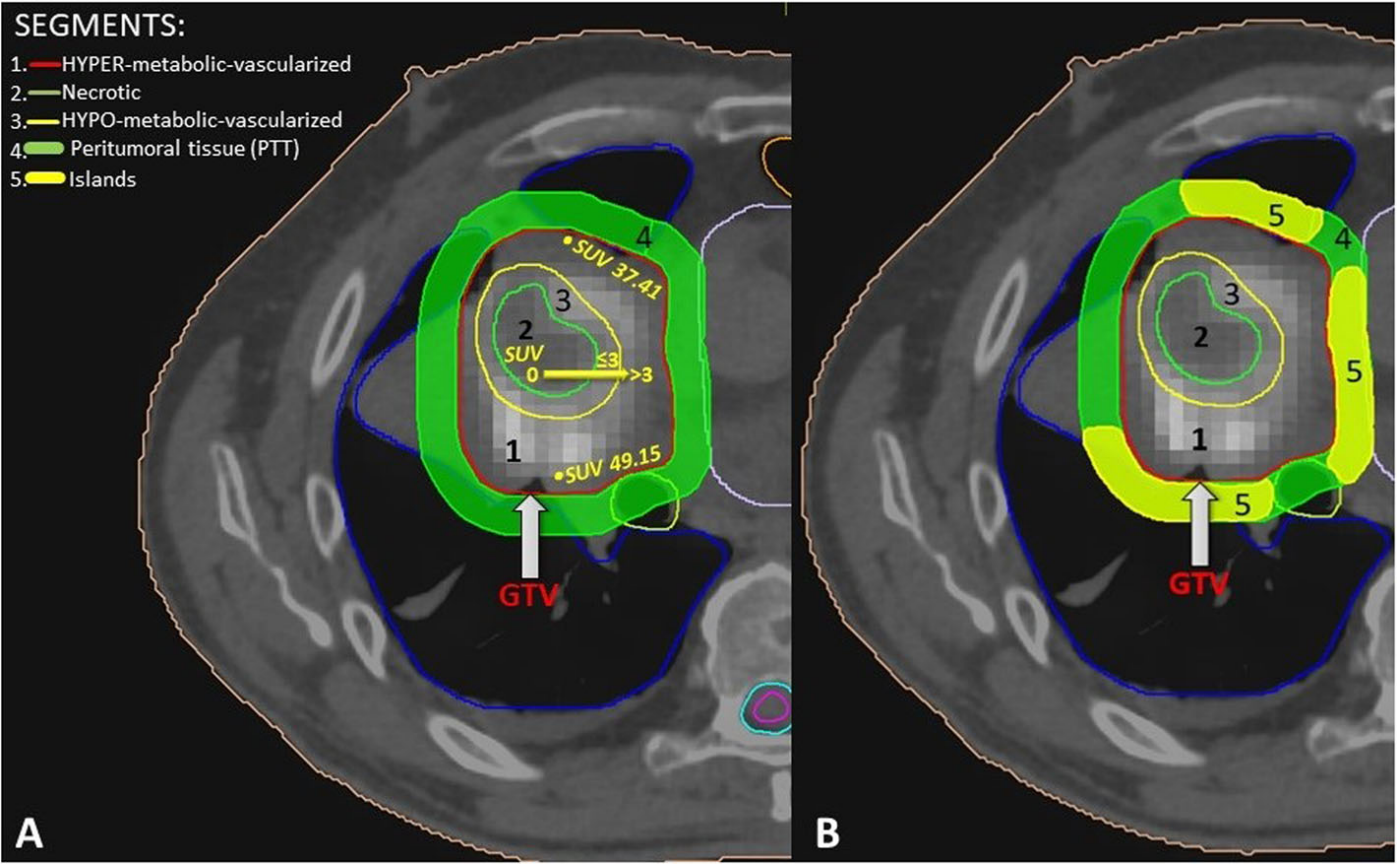
Target definition by segmentation of an unresectable bulky lung cancer (**a**): Segment 1- representing the contrast-enhanced (vascularized, “normoxic”) peripheral tumor segment, (GTV), Segment 2 - being the contrast-unenhanced (necrotic, “anoxic”) central tumor region, Segment 3- delineating the contrast-hypo-enhanced (hypovascularized, “hypoxic”) junctional tumor region as an up to a maximum of 5 mm junctional zone between the central-necrotic and the remaining peripheral-vascularized tumor segments, Segment 4 - representing peritumoral tissue; (**b**) Sparing of the peri-tumoral-surrounding immune microenvironment (thick green line) by creating as an organ at risk multiple “immune microenvironmental islands” (thick yellow line) in order to spare from radiation dose a significant volume (for ex. 2/3) of the tumor microenvironment, leaving it intact and functional

**SBRT-PATHY target definition** and **radiotherapy technique** have been previously described in detail [7]. We used a combination of CT and 18F-FDG-PET to define the hypovascularized (contrast-hypo-enhanced) and hypometabolic (SUV max < 3) tumor region representing the “hypoxic” segment between the central-necrotic and the remaining peripheral-vascularized tumor segments, which was then irradiated with 10Gy × 3 to the 70%-isodose line (Fig. 3).

### Patients

Eight patients with symptomatic, unresectable bulky tumors were prospectively treated between November 2017 and July 2019 with time-synchronized immune-guided SBRT-PATHY. All patients presented either distant metastases or regional metastatic lymph nodes that were not irradiated but followed for AE-induction. The treated patients’ main characteristics are summarized in Table 1. Two of eight patients previously received chemotherapy and immunotherapy, and both developed disease progression prior to SBRT-PATHY. No patient received any systemic treatment concomitant with SBRT-PATHY or before the second follow up after it. Two patients which previously received systemic treatment, continued with their treatment after 2 months post-SBRT-PATHY. The response evaluation was performed following the RECIST criteria at 1 month after the treatment by using CT and/or PET-CT, followed by repeated scans at month 2 and then every 3 months. Toxicity was evaluated using the CTCAE Criteria v5.0. All procedures performed in the present study were in accordance with the ethical standards. All the patients signed the informed consent. Present study has been registered by the local ethic committee under study number A 37/19.

**Table 1:** Patient and disease characteristics

FEATURES:	Number of patients (total 8):
GENDER:	
Male	5
Female	3
AGE (years):	
Mean	68.4
Range	57–80
ECOG PERFORMANCE STATUS:	
0–1	6
2–3	2
PRIMARY TUMOR SITE:	
Skin	1
Breast	1
Lung	6
HISTOLOGY:	
Adenocarcinoma (lung)	3
Adenocarcinoma (breast)	1
Squamous (lung)	3
Malignant melanoma	1
TREATED BULKY TUMOR SITE:	
Lung primary	6
Neck lymph node metastasis (skin melanoma)	1
Bone metastasis (breast)	1
BULKY TUMOR PATIENTS WITH DISTANT OLIGOMETASTASES:	3
Lung primary(1), malignant melanoma (1), Breast(1),	
BULKY TUMOR PATIENTS WITH LYMPH NODE METASTASES ONLY:	5
Lung primary(5)	
UNRESCTABLE BULKY TUMOR DIAMETER: mean/range (cm)	10.1/6.6–13.8
UNRESCTABLE BULKY TUMOR VOLUME: mean/range (cm^3^)	375.4/121.5–901.8
TARGETED BULKY TUMOR HYPOXIC SEGMENT: mean/range (cm^3^)	124.7/41.9–442.9
SYSTEMIC THERAPY (exclusively before SBRT-PATHY)	
Chemotherapy	1
Immunotherapy	1
SYMPTOMS related to bulky disease:	
Pain	6
Dyspnea	7
Cough	5
Haemoptysis	2
PATIENTS TREATED AT “MOST FAVOURABLE” DAY:	4
Lung primary: squamous(1) and adenocarcinoma(1)	2
Neck lymph node metastasis of malignant melanoma of the skin	1
Bone metastasis of breast adenocarcinoma	1
PATIENTS TREATED AT “LEAST FAVOURABLE” DAY:	4
Lung primary: squamous(2) and adenocarcinoma(2)	4

Abbreviations: *ECOG* Eastern Cooperative Oncology Group, *SBRT-PATHY* SBRT PArtial Tumor irradiation of the HYpoxic segment, *LLr* lower lobe right, *LLl* lower lobe left, *ULr* upper lobe right, *ULl* upper lobe left, *BTV* bystander tumor volume (hypoxic segment), *CHT* chemotherapy, *N.A.* not applicable

## Results

### Idiosyncratic immune cycle periodicity

The biomarker data analysis showed immune response fluctuations (Fig. 2) which were synchronized, following similar, regular frequency. The mean immune cycle duration was 7.3 days (range: 6.5–10). The average HS-CRP and LDH concentrations were 2.92 mg/dl (range: 0.13–7.35) and 492.8 (range: 163–1080), respectively.

### Clinical outcomes

**Fig. 4:**
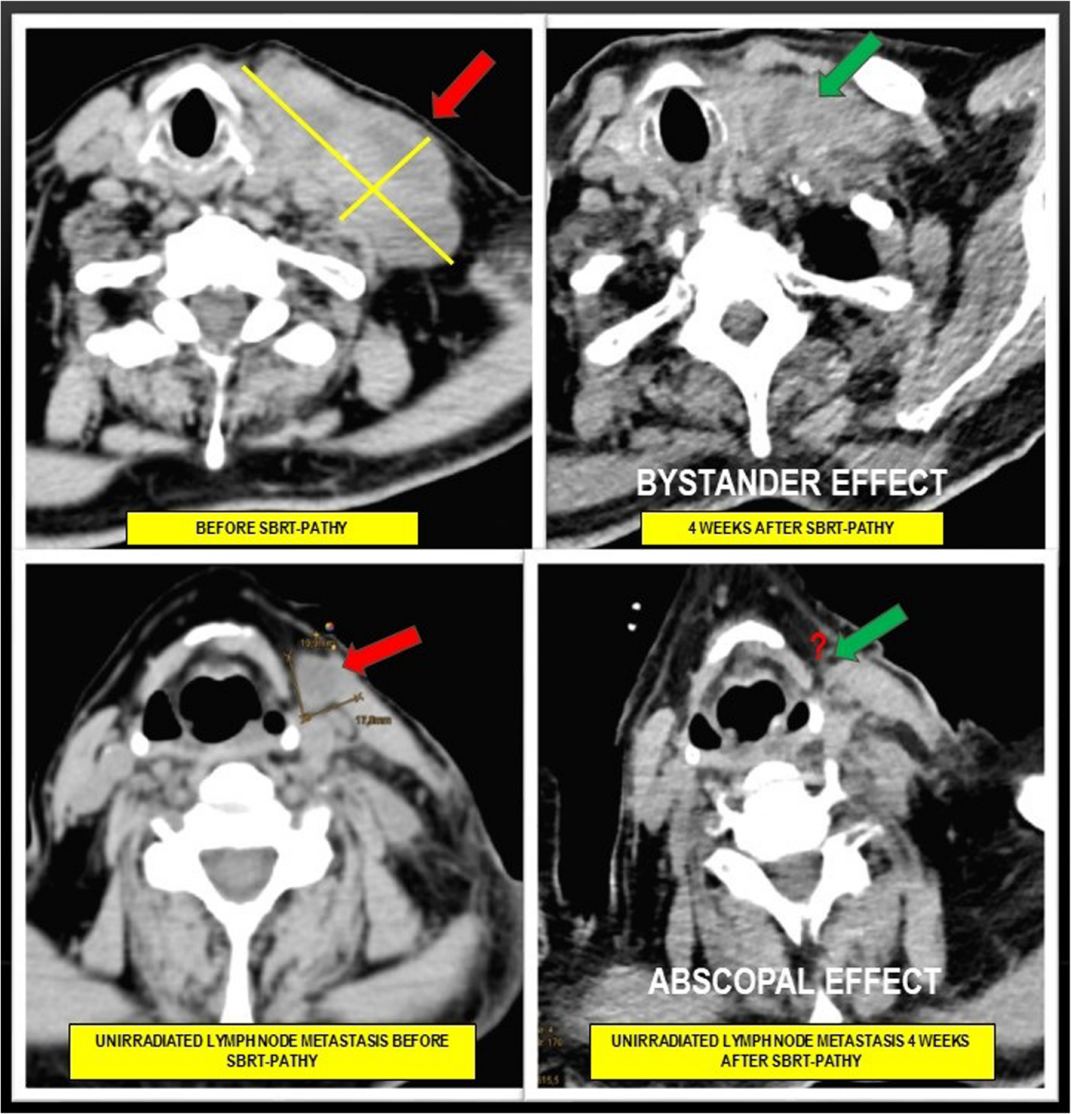
Induction of the bystander and abscopal effects by time-synchronized immune-guided SBRT-PATHY: Figure shows a very voluminous lymph node metastasis of the left neck in metastatic melanoma patient, with a maximum diameter of 10 × 7 cm before SBRT-PATHY (red arrow, upper image left), as well as one additional lymph node metastasis with a maximum diameter 2 × 2 cm that was not irradiated (red arrow, lower image left). A dramatic regression of the partially treated bulky lesion and also of unirradiated smaller lymph node metastasis was observed 4 weeks later (green arrows, upper and lower images right)

The median follow-up was 11.8 months (range: 4–22). At the time of analysis, one patient (treated at “most favourable” time-position) had died 22 months after SBRT-PATHY because of causes other than cancer. A significant BE (defined as a 30% or greater regression of partially treated bulky tumor) was observed in all four patients treated at “most favourable” time-position (including those two previously being in progression under systemic treatment) with an average tumor shrinkage of 100% (three complete responses (CR), one 80% tumor regression), while among those treated at “least favourable” time-position in two patients with an average tumor shrinkage of 35% (one CR, one 50% tumor regression, two stable bulky tumors reduced for 25%). Significant AE was observed in three patients treated at “most favourable” time-position (in lung and lymph node metastases, and in primary breast cancer) (Fig. 4), while in one among those treated at “least favourable” time-position (in lymph node metastases). Two patients with CR (one from each treatment group) were submitted two months after SBRT-PATHY to surgery, which confirmed pathologic CR at the level of the partially treated bulky tumor but also regional unirradiated lymph node metastases in both patients. Six out of eight patients were free from progression among which all four which were treated at “most favourable” day. In all these four patients was also achieved the symptom relief while among those treated at “least favourable” day it was observed in three patients. Four patients (two from each treatment group) experienced fatigue grade 1. No patient reported any late toxicity.

## Discussion

In addition to the partial tumor irradiation, sparing the loco-regional immune tumor microenvironment, the timing of radiotherapy in relation to the different phases of immune response could be the critical “missing link”. Recent evidences suggested that timing of therapy may influence clinical outcomes via immune modulation of the underlying immune response-suppression rather than direct tumor effects [18, 19]. In order to serially map the homeostatic immune response-fluctuations, we used Hs-CRP, LDH and LMR as surrogate of immune system interactions [15, 18, 19]. Since CRP synchronously rises and falls with initiation and termination of the immune response, we determined the start of the cycle as the “most favourable” day of the immune cycle “to release tumor antigen” by SBRT-PATHY, while the first day(s) of CRP fall as the “least favourable” (Fig. 2). This observations were accompanied with clinical outcomes suggesting significant BE/AE with the “most favorable day” approach.

The effectiveness in terms of BE/AE response rate of our novel concept could be explained by preserving the pre-existing/endogenous immune signaling in the non-irradiated tumor segment to be modulated by a sufficient threshold of cellular debris/antigen flow by SBRT-PATHY-induced cell damage. This could be seen as analogous to “radio-vaccination” event of manipulating the immunologic homeostatic balance of responsiveness and tolerance via endogenous inflammatory signals. In particular, in order to successfully modulate/disturb the homeostatic tumor immune-suppression, the immune system needs to be preserved as a real OAR.

## Conclusions

To our knowledge, this is the first evidence of a prospectively collected time-synchronized immune-guided radiotherapy in treating unresectable bulky tumor patients. We believe the right synchronization of radiotherapy with the homeostatically oscillating immune response may improve the probability of inducing BE/AE. Larger prospective trial on time-synchronized immune-guided SBRT-PATHY is ongoing at our institute.

## Data Availability

All data generated or analysed during this study are included in this published article and its supplementary information files. Any eventual further details on the data related to this study, are available from the corresponding author on reasonable request.
